# Elevated renal tissue oxygenation in premature fetal growth restricted neonates: An observational study

**DOI:** 10.1371/journal.pone.0204268

**Published:** 2018-09-20

**Authors:** Fieke Terstappen, Nina D. Paauw, Thomas Alderliesten, Jaap A. Joles, Daniel C. Vijlbrief, A. Titia Lely, Petra M. A. Lemmers

**Affiliations:** 1 Department of Obstetrics, Wilhelmina Children’s Hospital Birth Centre, University Medical Centre Utrecht, Utrecht University, Utrecht, the Netherlands; 2 Department of Neonatology, Wilhelmina Children’s Hospital Birth Centre, University Medical Centre Utrecht, Utrecht University, Utrecht, the Netherlands; 3 Department of Nephrology and Hypertension, University Medical Centre Utrecht, University Utrecht, Utrecht, the Netherlands; The University of Manchester, UNITED KINGDOM

## Abstract

**Background:**

Fetal growth restriction (FGR) is associated with an increased risk for kidney disease in later life. Studies reporting on early signs of renal disturbances in FGR are sparse and mostly include invasive measurements, which limit the possibility for early identification and prevention. We aim to investigate whether renal tissue oxygen saturation (rSO_2_) measured with near-infrared spectroscopy (NIRS) and the derived value fractional tissue oxygen extraction (FTOE) differ between premature FGR and control neonates in the first three days after birth.

**Methods:**

Nine FGR and seven control neonates born <32 weeks of gestation were included. FGR was defined as biometry <p10 combined with prenatal signs of placental insufficiency. Renal rSO_2_ was measured continuously with NIRS for 72 hours. FTOE was calculated as: (arterial saturation-rSO_2_)/arterial saturation. Renal artery blood flow (pulsatility and resistance index) was measured within 24 hours after birth. A linear mixed model approach was used (intercept ± slope = *r*) to analyze the NIRS parameters.

**Results:**

Renal rSO_2_ was higher in FGR neonates compared to controls (94% vs. 83%; p_group_ = 0.002). During the first three days after birth, renal rSO_2_ decreased in FGR neonates and increased in controls (*r* = -0.25 vs. *r* = 0.03; p_interaction_ = 0.001). Renal FTOE was lower in FGR neonates (0.02 vs. 0.14; p_group_ = 0.01) and increased slightly during three days after birth, while it remained stable in controls (*r* = 0.003 vs. *r* = -0.0001; p_interaction_ = 0.001). Renal artery blood flow was similar between groups.

**Conclusions:**

FGR neonate kidneys showed higher rSO_2_ as measured with NIRS and lower derived values of FTOE in the first three days after birth. We speculate that this was caused by either a reduced oxygen consumption due to impaired renal maturation or increased renal oxygen supply. How these observations correlate with short- and long-term renal function needs further investigation before renal NIRS can be implemented in screening and prevention in clinical practice.

## Introduction

Fetal growth restriction (FGR) is a condition in which the fetus fails to reach its genetic growth potential[1]. It increases the risk of perinatal morbidity and mortality, which is partly due to the higher likelihood of induction of premature delivery[2]. The major cause (70–80%) of FGR is placental insufficiency[3]. The pathophysiology of placental insufficiency is complex, and multiple factors including immunologic, angiogenic, and maternal genetic,—can lead to poor uteroplacental perfusion with reduced nutrient and oxygen supply to the fetus resulting in FGR[4].

As a response, blood flow preferentially distributes towards the fetal brain (brain-sparing). This occurs at the expense of perfusion to other organs. Studies report significant differences–however in contradicting direction—in renal blood flow of FGR fetuses compared to controls fetuses[5–7]. An adverse environment can negatively affect nephrogenesis, which completes in utero, and epigenetic programming of renal function[8,9]. This possibly contributes to the increased risk of hypertension and renal disease seen in FGR infants later in life[10].

Unfortunately, measuring renal function in premature neonates is challenging. Early neonatal creatinine and urea levels partly reflect maternal renal function, which limits the validity of these easily measured routine parameters during the first few days after birth. Therefore, studies on early signs of renal disturbances in FGR neonates are sparse and mostly include invasive measurements such as nephron counts or cumbersome measurements such as inulin clearance[11,12].

Near-infrared spectroscopy (NIRS) is a non-invasive bedside technique that is successfully used to continuously measure tissue oxygenation of the neonatal brain during the perinatal transition in preterm neonates. It measures the percentage of oxygenated hemoglobin of arterial, venous, and capillary blood in the tissue region under the sensor (rSO_2_) by the absorption difference between oxygenated and deoxygenated hemoglobin in the near-infrared spectrum of light. [13–16]

Better understanding of the adaptation of the neonatal kidneys to FGR could contribute to early identification of functional disturbances and prevention of hypertension and renal disease in later life. So far, tissue oxygenation has not yet been measured in FGR neonatal kidneys in comparison to an age-matched control group. The aim of this study is to measure renal tissue oxygen saturation with NIRS and the derived oxygen extraction to assess whether these variables differ between FGR and control neonates during the transitional phase. Either decreased oxygenation due to relatively low perfusion, and therefore low oxygen delivery, or increased oxygenation due to low solute reabsorption, and therefore low oxygen consumption, are conceivable as a mechanism for potentially observed differences.

## Methods

### Study population

This observational study was performed at the Wilhelmina Children’s Hospital in preterm neonates (gestational age [GA] <32 weeks) admitted to the neonatal intensive care unit from July 2016 until April 2017. Nine placental insufficiency-related cases of FGR were compared to seven controls. FGR was defined as estimated fetal weight (EFW) or abdominal circumference (AC) under the 10^th^ percentile. Exact percentiles for EFW were determined with the Hadlock formula using GA in weeks and days: 0.578 + 0.332*GA -0.00354*GA^2^ [17]. Placental insufficiency was defined as abnormal Doppler measurements in fetal middle cerebral artery (maximal velocity >p95), umbilical artery (absent or reversed end-diastolic flow or increased pulsatility index >p95), cerebral-placental ratio (decreased ratio in pulsatility index between umbilical artery and fetal middle cerebral artery <p5), uterine artery (notching or elevated resistance index p>95), or deflecting growth rate in 3 or more consecutive prenatal measurement points. The control group consisted of preterm neonates with normal growth for gestational age. Exclusion criteria were congenital disorders or multiple pregnancies. The Medical Ethical Committee of University Medical Centre Utrecht approved the study on 13-07-2016; protocol number 16-381C. Written informed consent was obtained from parents during pregnancy.

### Study protocol

The study protocol consisted of collecting clinical data and postnatal measurements from the medical records. Maternal comorbidities included cardiovascular disorders, diabetes, thyroid disorders and auto-immune disorders. Exact percentiles for birth weight and head circumference at birth were determined with Intergrowth-21^st^[18]. Neonatal complications included infant respiratory distress syndrome, intraventricular haemorrhage, sepsis and necrotizing enterocolitis. Treatment with mechanical ventilation during the period of admission was defined by intubation. The Score for Neonatal Acute Physiology with Perinatal Extension-II (SNAPPE-II) was calculated during the first 12 hours after birth as a predictor of mortality and morbidity[19]. Mean blood hemoglobin, arterial CO_2_ and CRP levels, as well as urine output (per kg body weight) were determined each day.

#### NIRS

Regional cerebral and renal oxygenations (rSO_2_) were measured continuously with a 2-wavelength (730 and 810 nm) NIRS for 72 hours after birth (INVOS 5100C, Medtronic, Boulder, CO). Sensors were placed as soon as possible after birth; a small adult sensor (SomaSensor SAFB-SM, Medtronic) was fixed on the forehead (left and right were alternated), and a neonatal sensor (OxyAlert CNN NIRSsensor) on the flank at the level of the left kidney. Sensor positioning was verified concomitantly with renal ultrasound. BedBase (in-house developed software) was used to simultaneously record SaO_2_, heart rate, systolic and diastolic blood pressure (SBP, DBP) by catheter and rSO_2_. One FGR neonate did not receive an intravascular catheter due to good clinical condition. Fractional tissue oxygen extraction (FTOE) was calculated by SignalBase (off-line version of BedBase) with the formula: FTOE = (SaO_2_ –rSO_2_)/SaO_2_. A decrease in FTOE can represent either an increase in oxygen supply or a decrease in oxygen consumption[20].

Artefacts were defined as 1) physiologically unexplained dips or peaks of at least 30% between two data points in rSO_2_ or in one of the accompanied parameters or 2) changes in rSO_2_ combined with severe deformed accompanying variables, alternated with missing data points, together suggesting neonatal movement[21]. After manually removing artefacts, 1-hour periods of consecutive high quality renal rSO_2_ data per 3-hour interval were selected. Most studies report tissue oxygenation data with 1-hour interval and therefore we also chose this to increase comparability. In cases of similarly high-quality data in a certain interval, the 1-hour period closest to the middle of the interval was selected to optimize time-based comparison between patients.

#### Renal ultrasound

A renal ultrasound of the right kidney was performed in seven FGR and six control neonates within 24 hours after birth (Aplio 400, Toshiba medical systems, Zoetermeer, The Netherlands, 7 MHz, convex probe). Pulsatility and resistance index in renal artery and renal length were measured 2–3 times and means were subsequently calculated. Renal ultrasound was not conducted in all patients due to unavailability of investigators and in one case due to poor neonatal condition. Most ultrasounds were performed by the same investigator (DCV) with exception of two ultrasounds.

### Statistical analysis

Statistical analysis was performed with IBM SPSS Statistics for Windows, version 24 (IBM Corp., Armonk, NY). To compare groups for clinical and ultrasound data, we used an independent t-test for continuous parametric data, Fisher exact test for categorical data, and Mann-Whitney test for continuous non-parametric data. Non-parametric data were expressed as mean ± standard error of the mean (s.e.m), counts (percentage), or median with interquartile range (IQR), respectively. Repeated neonatal measurements (hemoglobin, CRP, CO_2,_ urine production per body weight) were tested with two-way repeated ANOVA with Bonferroni’s post-hoc analysis to compare mean per day. We used a linear mixed-model statistical analysis for postnatal monitoring and NIRS parameters with individuals as a random effect nested within the groups and compound symmetry as (repeated) covariance type. We did not correct for factors or covariates due to the small sample size. The group effect (p_group_) and whether the groups behaved differently over time (*r =* slope per 3-hour interval; p_interaction_) were investigated. A two-sided p-value < 0.05 was considered significant.

## Results

### Baseline characteristics

No maternal comorbidities were present in the control group, while in the FGR group, one pregnancy was complicated by a thyroid disorder and one by pre-existing hypertension (Table 1). Seven of the FGR neonates’ mothers experienced pre-eclampsia. More antihypertensive drugs and fewer antibiotics were used in pregnancies complicated by FGR than in controls. Five of the FGR cases were predicted to be severely growth restricted (EFW<p3), two showed asymmetric growth restriction and seven showed an abnormal ratio in pulsatility index between umbilical artery and middle cerebral artery. Neonatal baseline characteristics are presented in Table 2; note that the gestational age was similar between groups at the time that regional oxygenation was measured with NIRS. Hemoglobin levels per day were higher in FGR vs. control neonates, but remained within reference ranges[22]. CRP, CO_2_ levels and urine production per body weight did not differ significantly between groups over time.

**Table 1:** Maternal and pregnancy characteristics.

	FGR (n = 9)	Control (n = 7)	p-value
**Maternal**			
Age in years	29.7 ± 1.3	29.7 ± 1.6	0.98
BMI	25.9 ± 1.7	24.0 ± 1.9	0.47
Ethnicity: Caucasian	4 (44.4)	7 (100)*	**0.03**
Comorbidities	2 (22.2)	0 (0)	0.48
**Pregnancy**			
Preeclampsia	7 (78)	0 (0)**	**<0.01**
Smoking	4 (44)	2 (29)	0.63
Caesarean section	9 (100)	4 (57)	0.06
**Medication during pregnancy**			
MgSO_4_	8 (89)	6 (86)	1.00
Antihypertensive drugs	7 (78)	1 (14)*	**0.04**
Antidepressant	0 (0)	1 (14)	0.44
Immuno-depressant	1 (0)	2 (29)	0.18
Insulin	0 (0)	1 (14)	0.44
Antibiotics	2 (22)	6 (86)*	**0.04**
Paracetamol	3 (33)	2 (29)	1.00
**Prenatal ultrasound**			
GA at ultrasound measurement	29.6 ± 0.6	26.3 ± 0.8**	**<0.01**
EFW percentile < p3	5 (56)	0 (0)	0.11
Asymmetric FGR	2 (22)	0 (0)	1.00
UA PI	1.70 ± 0.11	N/A	N/A
UA PI > p95	6 (67)	N/A	N/A
MCA V_max_ (cm/s)	39.0 ± 1.9	N/A	N/A
MCA V_max_ > p95	1 (17)	N/A	N/A
UA/MCA PI ratio > p95	7 (78)	N/A	N/A

Data shown as mean ± s.e.m. or n (%) and analysed with respectively independent t-test or Fisher exact test. Asymmetric FGR is defined as head circumference/abdominal circumference ratio >p95. Abbreviations: BMI, body mass index; EFW, estimated fetal weight; FGR, fetal growth restriction; GA, gestational age; MCA, middle cerebral artery; MgSO_4,_ magnesium sulphate; N/A, not available; PI, pulsatility index; UA, umbilical artery; V_max_, maximal velocity.

**Table 2:** Baseline characteristics of neonates and neonatal measurements.

	FGR (n = 9)	Control (n = 7)	p-value
**Neonate**			
GA at birth in weeks	30.6 ± 0.5	29.1 ± 1.0	0.18
Gender: male	7 (78)	4 (57)	0.60
BW in grams	1106 ± 71	1411 ± 176	0.10
BW percentile	8 (3–17)	74 (70–81)	**0.001**
HC at birth in cm	26.3 ± 0.7	26.8 ± 1.0	0.67
HC at birth percentiles	16.1 ± 6.1	44.9 ± 9.7	**0.02**
**Medication**			
Incorporated antenatal steroids (n, %)	8 (89)	7 (100)	1.00
Postnatal steroids (n,%)	0 (0)	1 (14)	0.44
Antibiotics (n,%)	6 (67)	7 (100)	0.21
Surfactant treatment (n,%)	3 (33)	2 (29)	0.60
Ductus arteriosus treatment (n,%)	0 (0)	1 (14)	0.40
Inotropine (n,%)	0 (0)	0 (0)	1.0
**Neonatal health**			
*Umbilical cord blood*			
pH arterial	7.26 ± 0.03	7.33 ± 0.03	0.17
Base excess	-2.67 ± 0.88	-6.00 ± 1.53	0.13
*Apgar score <5*			
1 min	1 (11)	3 (43)	0.26
5 min	0 (0)	3 (43)	0.06
SNAPPE-II score	5 (0–16)	33 (9–40)	0.09
NICU admission in days	13 (10–20)	17 (16–87)	0.08
Mechanical ventilation	2 (22)	4 (57)	0.30
*Neonatal complications*	1 (11)	4 (57)	0.11
IRDS	1 (11)	2 (29)	
Sepsis	0 (0)	1 (14)	
IVH	0 (0)	2 (29)	
**Neonatal measurements**			
*CRP in mg L*^*-1*^			0.15
Day 1	2.4 ± 0.7	1.7 ± 1.2	>0.99
Day 2	4.4 ± 1.2	2.5 ± 1.7	0.92
Day 3	4.8 ± 2.4	1.5 ± 0.5	0.23
*Hemoglobin in mmol L*^*-1*^			**<0.001**
Day 1	12.0 ± 0.3	9.8 ± 0.4	**<0.001**
Day 2	11.4 ± 0.3	9.3 ± 0.3	**<0.001**
Day 3	11.1 ± 0.4	10.1 ± 0.2	0.07
*CO*_*2*_ *in mmHg*			0.76
Day 1	42.6 ± 2.8	43.9 ± 3.1	>0.99
Day 2	42.1 ± 1.2	37.5 ± 2.8	0.52
Day 3	41.7 ± 1.5	42.1 ± 1.4	>0.99
*Urine output in ml/kg BW*			0.30
Day 1	87.5 ± 11.2	92.1 ± 12.5	>0.99
Day 2	103.5 ± 14.8	110.5 ± 5.5	>0.99
Day 3	114.2 ± 7.8	141.2 ± 13.0	0.32

Data shown as mean ± s.e.m., n (%) or median (IQR) and analysed with respectively independent t-test, Fisher exact test, or Mann-Whitney test. The neonatal measurements were tested with two-way repeated measures ANOVA with Bonferroni’s post-hoc analysis to compare per day.

Abbreviations: BW, birth weight; CO_2,_ carbon dioxide; CRP, C-reactive protein; FGR, fetal growth restriction; GA, gestational age; HC, head circumference; IRDS, infant respiratory distress syndrome; IVH, intraventricular haemorrhage; NICU, neonatal intensive care unit; SNAPPE-II, score for neonatal acute physiology with perinatal extension-II

### Renal and cerebral rSO_2_ and FTOE

**Fig 1:**
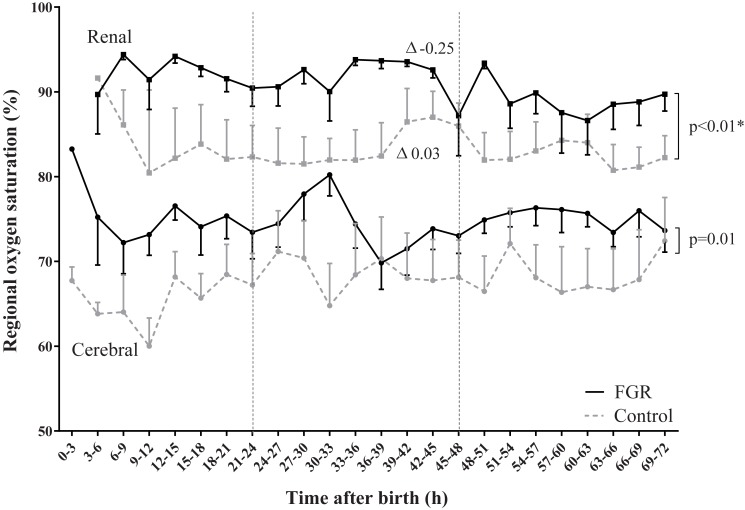
Regional oxygen saturation (rSO_2_). Renal and cerebral rSO_2_ are elevated in fetal growth restriction (FGR) vs. control neonates and over time groups behaved differently in renal rSO_2_. Significant group differences are shown and * indicates whether the groups behaved differently over time (p_interaction_<0.05). ∆ indicates the slope per 3 hours interval.

Renal rSO_2_ was higher in FGR vs. control neonates (Fig 1 and Table 3*)*. During the first three days after birth, the groups behaved differently: renal rSO_2_ decreased in FGR neonates but increased in controls. Cerebral oxygenation was higher in FGR vs. control neonates. Cerebral rSO_2_ in both groups was similarly stable over time.

**Table 3:** Variables measured with or derived from NIRS per neonatal group.

		Intercept	95% CI	p_group_	*r*	95% CI	p_interaction_
**Cerebral rSO_2_ (%)**	FGR	74.4	70.1; 78.8	**0.01**	0.04	-0.14; 0.21	0.40
	Control	65.8	61.0; 70.7		0.14	-0.04; 0.34	
**Renal rSO_2_ (%)**	FGR	93.7	88.9; 98.6	**0.002**	-0.25	-0.38;-0.11	**0.01**
	Control	82.7	77.7; 87.7		0.03	-0.13;0.20	
**Cerebral FTOE**	FGR	0.19	0.13; 0.24	**0.005**	0.001	-0.001; 0.003	0.10
	Control	0.31	0.25; 0.37		-0.001	-0.003; 0.001	
**Renal FTOE**	FGR	0.02	-0.02; 0.07	**0.004**	0.003	0.002; 0.004	**0.001**
	Control	0.14	0.08; 0.19		-0.0001	-0.003; 0.001	

*r* = slope per 3 hour interval. *

Abbreviations: CI, confidence interval; FGR, fetal growth restriction; FTOE, fractional tissue oxygen extraction; NIRS, near-infrared spectroscopy; rSO_2,_ regional oxygenation

**Fig 2:**
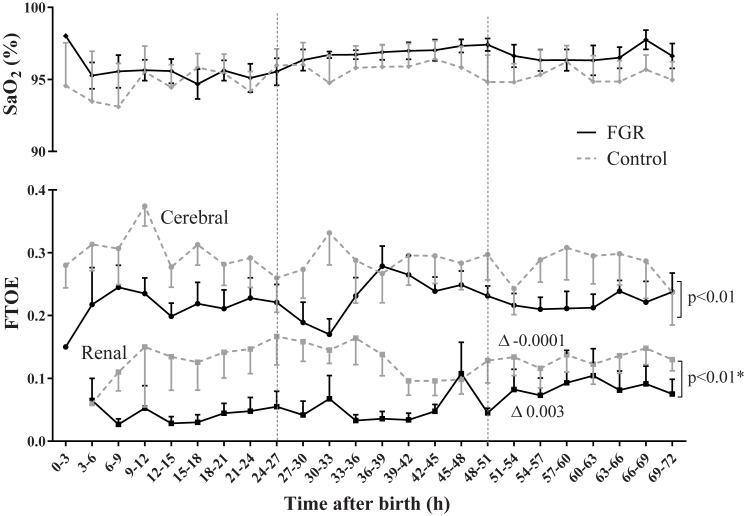
Arterial saturation (SaO_2_) and fractional tissue oxygen extraction (FTOE). Arterial saturation was similar between groups and over time. Renal and cerebral FTOE was lower in FGR compared to controls. Renal FTOE, but not cerebral FTOE, behaved differently over time. Significant group differences are shown and * indicates whether the groups behaved differently over time (p_interaction_<0.05). ∆ indicates the slope per 3 hours interval. Data shown as mean ± s.e.m.

Renal FTOE was lower in FGR neonates vs. controls (Fig 2). Renal FTOE increased slightly in FGR neonates during the first three days after birth, while it remained stable in controls. Cerebral FTOE was lower in FGR vs. control neonates and both groups remained stable during the first three days after birth. Arterial oxygen saturation was similar between groups (Table 4).

**Table 4:** Bedside monitoring variables.

		Intercept	95% CI	p_group_	*r*	95% CI	p_interaction_
**HR (bpm)**	FGR	135	128; 142	0.07	0.93	0.73; 1.12	**<0.01**
	Control	145	137; 153		0.09	-0.13’0.31	
**SBP (mmHg)**	FGR	45.4	42.1; 48.6	0.48	0.45	0.34; 0.56	0.61
	Control	43.7	40.2; 47.2		0.40	0.28; 0.52	
**DBP (mmHg)**	FGR	31.9	28.1; 35.8	0.27	0.26	0.16; 0.37	0.25
	Control	28.9	24.7; 33.0		0.17	0.06; 0.29	
**SaO_2_ (%)**	FGR	95.4	93.5; 97.3	0.73	0.07	0.03; 0.11	0.44
	Control	94.9	92.8; 97.0		0.05	0.0004; 0.09	

*r* = slope per 3-hour interval.

Abbreviations: bpm, beat per minute; FGR, fetal growth restriction; HR, heart rate; SBP, systolic blood pressure; DBP, diastolic blood pressure; SaO_2_, oxygen saturation

### Blood pressure and heart rate

**Fig 3:**
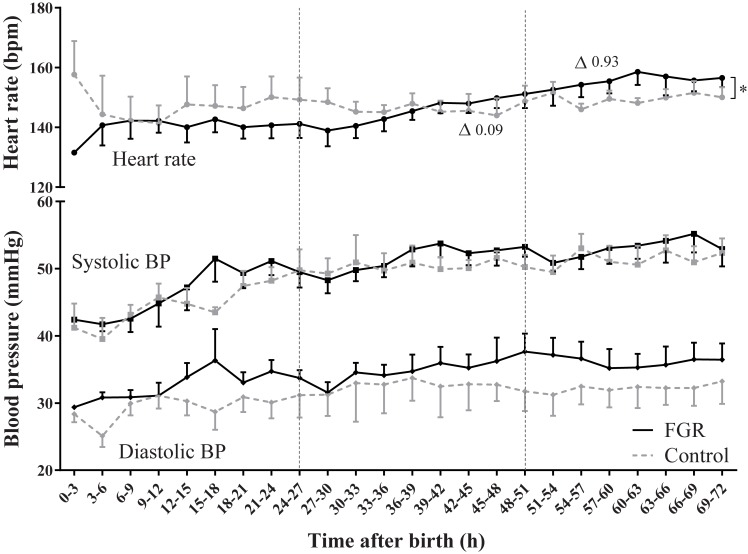
Heart rate and systolic and diastolic blood pressure (SBP and DBP). Heart rate and systolic and diastolic blood pressure were similar between groups. During the three days after birth, only heart rate increased significantly more in FGR compared to controls. Significant group differences are shown and * indicates whether the groups behaved differently over time (p_interaction_<0.05). ∆ indicates the slope per 3 hours interval. Data shown as mean ± s.e.m.

Heart rate was similar between groups but increased significantly over time in FGR only (Fig 3 and Table 4*)*. SBP and DBP were similar between groups and increased to the same extent in both groups over the 72 hours after birth.

### Renal ultrasound in first day after birth

The renal ultrasound was performed at a similar mean time after birth (FGR: 15.0 ± 2.9 hours after birth vs. control 13.9 ± 2.7 hours after birth; p = 0.80). No significant differences were found in pulsatility index and lower resistance index of the renal artery or kidney length in FGR vs. control neonates (Table 5).

**Table 5:** Postnatal ultrasound of renal artery blood flow and kidney length.

	FGR (n = 7)	Control (n = 6)	p-value
**Pulsatility index**	2.25 ± 0.39	2.06 ± 0.34	0.71
**Resistance index**	0.86 ± 0.05	0.93 ± 0.10	0.53
**Kidney length (mm)**	31.6 ± 1.3	32.5 ± 2.5	0.71

Renal artery blood flow measured within 24 hours after birth.

Data shown as mean ± s.e.m. and analysed with independent t-test. Abbreviation: FGR, fetal growth restriction.

## Discussion

The preterm FGR neonates in our study showed higher renal and cerebral rSO_2_ with NIRS during the first three days after birth compared to preterm controls. Additionally, we showed that both renal and cerebral FTOE was lower in FGR neonates. Renal artery blood flow did not differ between groups.

To the best of our knowledge, this is the first study in which renal tissue oxygenation was compared between FGR neonates and controls during the transition from intrauterine to extrauterine life. Tanis et al. showed higher renal FTOE in early FGR neonates compared with late FGR neonates (but control neonates were not included in this study) and found that fetal brain-sparing signs were correlated with a low cerebral-renal ratio after birth, supporting the concept of preferential redistribution of blood after birth[16]. Similar to our findings, other studies also failed to detect differences in renal blood flow between the two groups[23,24].

Higher cerebral rSO_2_ and lower cerebral FTOE in FGR neonates correspond to earlier findings, confirming the validity of the NIRS technique[25,26]. These differences in cerebral oxygenation have been attributed to an increased cerebral blood flow, caused by vasodilation in order to maintain cerebral oxygen supply—an adaptation to the adverse prenatal FGR environment (as continuation of brain-sparing)[25,26]. Despite similar findings of increased tissue oxygenation in the kidney and the brain, this is not necessarily caused by the same mechanism as characteristics of blood flow regulation in brain and kidney are fundamentally different[27].

Increased renal tissue oxygenation might be caused by decreased renal arteriovenous shunting, decreased oxygen consumption in the kidneys, or by increased renal blood flow, which is most often the case[28]. However, we found that renal blood flow did not significantly differ between groups. Increased oxygen supply could also be caused by systemic vasodilation, increased arterial saturation, elevated levels of hemoglobin or increased cardiac output[28]. However, blood pressure was similar between groups and heart rate was slightly reduced rather than increased during the first three days after birth. While hemoglobin was within the reference range, levels were significantly higher in FGR vs. control neonates, although this difference decreased over time. Erythropoiesis is accelerated in growth restricted fetuses, presumably as a compensation for the decreased placental oxygen supply to the fetus[25,26]. Due to a small sample size, we could not perform a correlation between the renal or cerebral oxygenation and hemoglobin levels in our study. Ishii et al. did not observe a correlation between the level of hemoglobin and cerebral oxygenation in small for gestational age neonates[26]. In addition, fractional fetal hemoglobin is higher in FGR neonates[26]. As fetal hemoglobin has slightly different absorption curves in the near-infrared range, this could also contribute to the higher rSO_2_ levels[29]. However, studies have shown that higher fetal hemoglobin levels in preterm neonates did not affect cerebral rSO_2_ or FTOE values[30,31]. Whether this also applies to renal rSO_2_ is still unknown.

On the other hand, the increased renal tissue oxygenation in FGR neonates that we observed may be caused by decreased oxygen consumption. Renal oxygen consumption depends mainly on sodium reabsorption and indirectly on glomerular filtration rate (GFR), renal blood flow and systemic vasodilation[28]. As the latter two are similar between the groups, we speculate that the decreased oxygen consumption is most likely caused by a decrease in the absolute level of sodium reabsorption, possibly secondary to a low GFR. Correspondingly, an increased fractional sodium excretion has been detected in FGR neonates on the first day after birth[32]. This speculation is in line with the reduced number of nephrons that has been reported following FGR in both human and animal studies[8,11,33–35]. Teleologically, low sodium reabsorption would be necessary to prevent volume overload in the setting of low GFR.

Differences in baseline characteristics may also have influenced renal tissue oxygenation. Antihypertensive drug use in pregnancies complicated by FGR could affect hemodynamic parameters and thereby tissue oxygenation. Maternal beta-blockers intake could result in reduced renin release, reduced heart rate–as observed in this study–and vasodilatation. While theoretically this could result in lower renal or cerebral rSO_2_, Thewissen et al. did not find a correlation between labetalol use and cerebral tissue oxygenation[36].

This study has several strengths. This is the first study comparing renal tissue oxygenation measured with NIRS between preterm FGR and preterm control neonates. NIRS parameters were measured continuously during the first three days after birth in contrast to other studies, in which tissue oxygenation was only measured intermittently with NIRS. Aside from renal NIRS, we studied renal artery blood flow, arterial blood pressure, heart rate, and arterial saturation. In addition, we used prenatal ultrasound measurements to clearly define placental insufficiency phenotype in our study population.

We acknowledge some limitations. The number of participants is limited. Despite the small sample size, a highly significant difference in renal oxygenation was found. However, a type II error might have occurred with renal artery blood flow. As a result, caution regarding the conclusion on renal artery blood flow limits us in leaning towards one of our two speculations on underlying mechanisms. We were also unable to adjust for covariates or different baseline characteristics that might have been of influence in the linear mixed model. Another limitation is that renal NIRS parameters have not yet been linked to renal function parameters, which is difficult due to the lack of renal function measurements especially during the first three days after birth. Persistent ductus arteriosus might have influenced our results, as previous studies report abnormal renal Doppler patterns [37, 38] and reduced cerebral oxygenation around 72 hours after birth in preterm neonates with persistent ductus arteriosus [39,40]. An ultrasound to assess left ventricular cardiac output and persistent ductus arteriosus (with shunt direction) at the time of renal ultrasound would have helped to better interpret the results. In addition, repeat renal artery Doppler measurements during the these first three days after birth could have provided additional insight. As the neonatal sensors are relatively large for the FGR neonates, they may also have registered organs adjacent to the kidney, such as adrenal glands[41]. Based on previous literature we selected 1-hour intervals to increase comparability. Mintzer *et al*. showed that shorter selected intervals of tissue oxygenation measured with NIRS reduces baseline variability[42]. Although a different interval than 1 hour might slightly influence our results, it is unlikely that shorter intervals with reduced variability would result in a substantially different outcome of our study. Future studies to the exact influence of different selected time periods would be of interest.

While we speculate that increased renal rSO_2_ in FGR is caused by impaired renal maturation, no study has correlated NIRS parameters at birth with renal function at birth or in later life. Unfortunately, the restricted measurements options for renal function in the neonatal period (glomerular filtration rate, urea, and creatinine were not measured in the included neonates during their first three days of admission) limit us in studying this correlation and make fractional sodium excretion the best measurement option. A larger study including interesting renal function measurements—especially fractional electrolyte excretions—is of importance to further explore our speculation on decreased oxygen consumption. Follow-up studies should identify whether renal rSO_2_ correlates with renal function in the neonatal period (fractional sodium excretion), and with renal function (GFR, creatinine clearance) and blood pressure in childhood and adulthood.

In conclusion, elevated tissue oxygenation and lower oxygen extraction is observed in kidneys of FGR neonates during the first three days after birth. This can be caused by decreased oxygen consumption in the kidneys and/or increased oxygen supply. To make a better distinction between those two potential underlying mechanisms, this study should be performed with a larger sample size and, in particular, include repeated renal artery Doppler measurements and, although difficult to achieve, fractional electrolyte excretion. How these findings correlate with renal function on the short- and long-term needs further investigation before renal NIRS can be implemented in clinical practice. This applies to both screening (follow-up renal measurements of neonates potentially at risk) and prevention (avoidance of second hits such as nephrotoxic agents or high salt diets; monitoring of potential improvement after in utero strategies to prevent FGR and renal impairment).
